# Neonatal mice resist *Plasmodium yoelii* infection until exposed to para-aminobenzoic acid containing diet after weaning

**DOI:** 10.1038/s41598-020-79703-2

**Published:** 2021-01-08

**Authors:** Marcela Parra, Jiyeon Yang, Megan Weitner, Mustafa Akkoyunlu

**Affiliations:** 1 Center for Biologics Evaluation and Research, Division of Bacterial Allergenic and Parasitic Diseases, US Food and Drug Administration, Silver Spring, MD USA; 2 DBPAP, US FDA, CBER, 10903 New Hampshire Ave., Building 52/72, Room, 5214, Silver Spring, MD 209903 USA; 3 Present Address: Viral & Rickettsial Diseases Department (NMRC VRDD), Naval Medical Research Center, Silver Spring, MD USA

**Keywords:** Immunology, Microbiology, Pathogenesis

## Abstract

We developed a newborn (NB) mouse *Plasmodium yoelii* NL infection model to study malaria in early age. Surprisingly, the onset of parasitemia in *P. yoelii* challenged NB mice was delayed compared to adults and coincided with the weaning date when weanlings switched from maternal milk to normal chow diet. Also, compared to adult mice, parasitemia resolved much later (48 days vs 20 days post challenge) and the peak parasitemia was twice as high in weanlings. Concurrently, weanlings’ germinal center reaction was delayed and diminished compared to adult mice. Maternal milk is deficient in para-aminobenzoic acid (PABA), which is required for de novo folate synthesis by *Plasmodium*. Suggesting a possible role for the protection afforded by PABA-deficient maternal milk, mice fed with a PABA-deficient diet after the weaning continued to control parasitemia. Despite the reduced parasitemia, these mice developed robust T follicular helper (Tfh) responses and were protected from a second *P. yoelii* challenge. The NB malaria model provides mechanistic insight into the human infant malaria manifestations where a diet solely based on breast-feeding reduces the incidence of severe malaria in infants. NB mice experiments also support further studies to investigate dietary PABA restriction in the management of severe malaria in infants.

## Introduction

Malaria continues to be a threat for a big segment of the world population. In 2018 the World Health Organization (WHO) reported 228 × 10^6^ infections and 405,000 deaths, with a substantial number of the cases occurring in African children under 5 years of age^1^. *Plasmodium falciparum* is responsible for the majority of malaria deaths globally, although *P. vivax*, *P. ovale*, *P. malariae* are also causes of substantial morbidity^2^. Among the pediatric malaria cases those who suffer from malaria during the first 6 months of life manifest the disease differently than the older children^3,4^. Generally, the incidence of malaria during infancy is considered to be lower than later in life^4–6^, although the advancements in molecular diagnostics helped determine the persistence of parasitemia in asymptomatic infants^7^. In addition to lower incidence of disease, those who are infected tend to have low levels of parasitemia^8^. In infants, severe malaria can manifest itself as cerebral malaria, severe anemia and respiratory distress among others^2,9^. Although the exact reasons are not elucidated, the development of severe malaria appears to be associated with the absence of anti-malarial antibodies^10,11^.

The low incidence of disease in infants has been attributed to several factors but limited experimental data have emerged in support of these factors. For example, early reports recognized maternal antibodies as protective mostly because of the decreased incidence of infection until the waning of maternal antibodies at about 9 months after birth^12–14^. However subsequent studies not only questioned the value of maternal antibodies, but some also suggested an association between maternal antibodies and increased risk of infection^13,15^. Another factor implicated in the lower incidence of malaria in early life is the presence of fetal hemoglobin (HbF). Early studies suggested that *P. falciparum* growth is arrested in cord blood erythrocytes due to HbF^16,17^. Subsequent reports demonstrated that Hbf does not inhibit *P. falciparum* growth in infant red blood cells^18,19^. The dietary elements of neonatal period can also be contributing to the protection of infants from malaria^20^. Maternal milk is shown to contain lactoferrin and IgA antibodies directed against parasite antigens, both of which can control parasite growth, at least in vitro^21^. Additionally, the deficiency of maternal milk of p-aminobenzoic acid (PABA) is suggested to be providing additional protection of infants from malaria infection^3,13,22^. This hypothesis is based on the fact that malaria needs PABA for de novo folate synthesis and malaria parasite cannot establish infection in adult mouse fed on PABA-deficient diet^23–25^.

Here, we established an infant mouse *P. yoelii* infection model to study the disease progression and host response to malaria. We found that malaria outcomes in young mice differ in parasitemia kinetics and magnitude from the adult mice. The main reason for the difference in disease outcome appears to be due to the suppression of parasitemia associated with limited availability of PABA in suckling mice until the switch of diet to NCD at weaning. Continuation of milk-based diet or PABA-deficient NCD maintained the low parasitemia until the mice mounted protective immune response and resolved parasitemia. Underscoring the role of PABA in *Plasmodium* survival, removal of PABA from the diet of already infected weanlings effectively reduced parasite load. Taken together, NB mouse *P. yoelii* infection model provides an opportunity to study human infant malaria because the infection outcome recapitulates many features of infant malaria in endemic areas, including the reduced incidence of severe malaria observed in infants fed with maternal milk^20^.

## Results

### *Plasmodium yoelii* challenged newborns control parasitemia until after they are weaned

**Figure 1:**
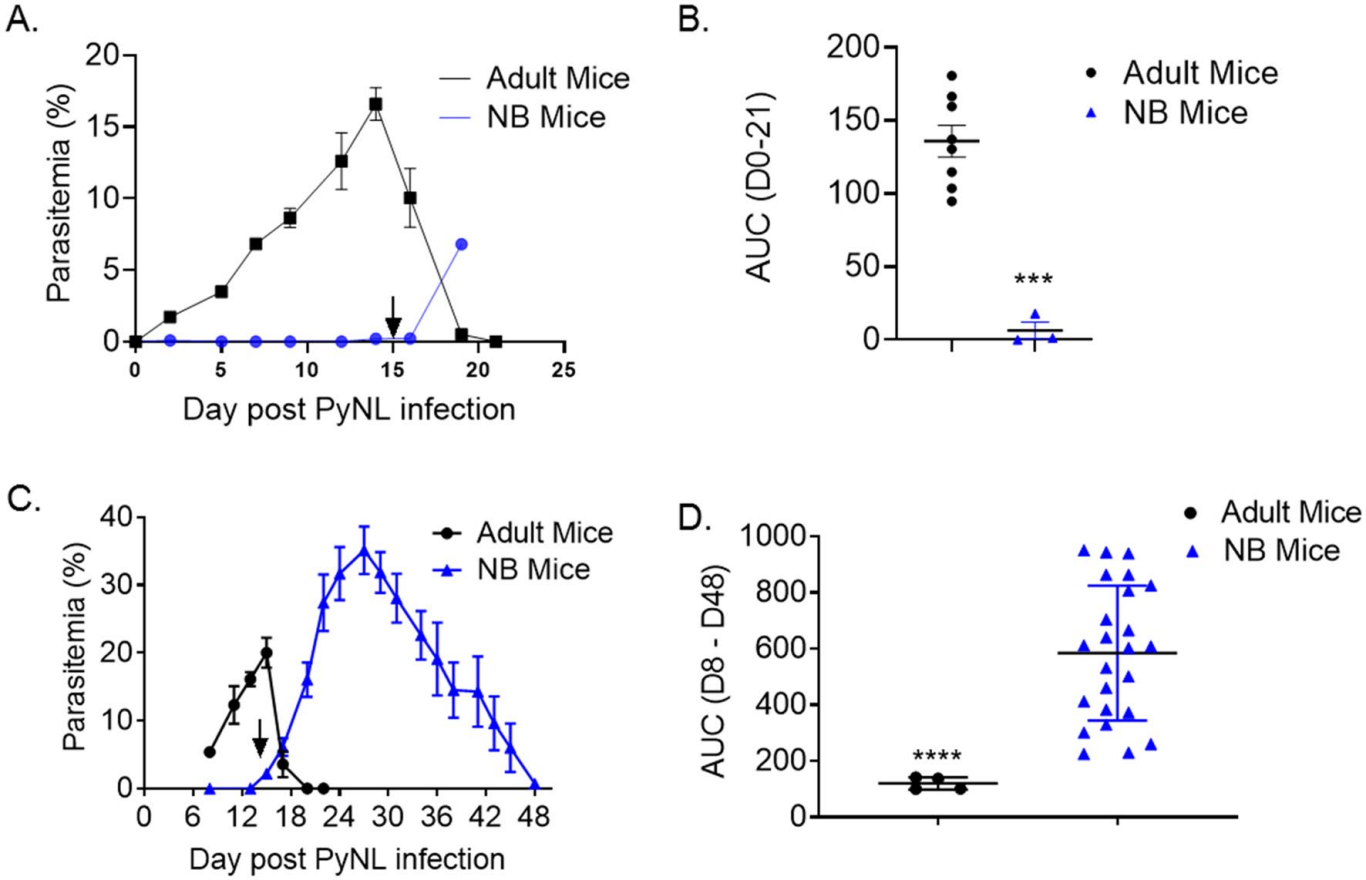
Latency is extended and parasitemia worsened in the NB mice infected with PyNL when compared to adult mice. (**A**) Twenty 6 to 7-days old NB and 8 7- to 8-weeks old adult C57BL/6 mice were infected with 1 × 10^6^ PyNL parasites. Parasitemia levels were evaluated by blood smear every 2 to 3 days after challenge until parasitemia clearance. NB mice were weaned at 21 days of age (15 days post infection black arrow). (**B**) AUC of parasitemia burden between the days 0 to 21 post infection shown in panel (**A**) are plotted. (**A**, **B**) Cumulative data (mean ± SEM) from two experiments are shown. (**C**) Twenty-four 6- to 7-days old NB and 4 7- to 8-weeks old adult C57BL/6 mice were infected with 1 × 10^6^ PyNL parasites. Parasitemia levels were evaluated by blood smear on indicated time points. NB mice were weaned at 21 days of age (black arrow). (**D**) AUC of parasitemia burden between the days 8 to 48 post infection shown in panel C are plotted. (**C**, **D**) Cumulative data (mean ± SEM) from three experiments are shown. Student’s t-test was used to compare the AUC between groups. ***p < 0.001.

Unique futures of neonatal immune system render them susceptible to infectious diseases^26^. Yet, the clinical reports clearly indicate that neonates and infants experience malaria disease less than children at older ages^4–6^. At the same time, infants who develop malaria manifest more severe disease compared to adults^2,9^. To investigate the biological basis of neonatal responses to malaria infection, we established a NB mouse *P. yoelii* infection model. We challenged 6- to 7-days old mice with 1 × 10^6^ PyNL parasites and assessed the development of malaria infection by monitoring parasitemia. Adult mice challenged with the same number of parasites served as control. As reported previously^27^, parasitemia was detected as early as 2 days post-challenge (1.7%) and peaked at day 14 post-challenge to 16.6% in adult mice (Fig. 1A). Adult mice parasitemia was resolved by day 21 post-challenge. In sharp contrast, parasitemia was not detected in NB mice until day 14 post-challenge. Weanlings’ parasitemia increased to 6.81% on day 19 post-challenge when the mice were euthanized for serum collection. Parasitemia burdens quantified as area under curve (AUC) until day 21 when adult mice cleared parasitemia also showed a big difference between the weanlings and adult mice (Fig. 1B). We then repeated the PyNL challenge experiments to follow weanling parasitemia for an extended duration. As in the first challenge experiment, adult mice parasitemia peaked at 2 weeks (day 15) after PyNL challenge (20.02%) (Fig. 1C). The kinetics of parasitemia in challenged NB mice was similar to that in the short term follow-up experiment (Fig. 1A). In challenged NB mice, the first smear positive sample (1.05%) was on day 15 post-challenge. More importantly, the parasitemia gradually increased thereafter, reaching a peak parasitemia percentage of 35.11% at day 27 post-challenge. Parasitemia burdens quantified as AUC until day 48 when weanlings cleared parasitemia also showed a significant difference between the weanlings and adult mice (Fig. 1D). The parasitemia in weanlings also persisted much longer than in adults. Weanlings became smear negative at 48 days-post challenge, which was 28 days after the clearance of parasitemia in adult mice.

Newborn *P. yoelii* challenge experiments clearly showed that the kinetics and magnitude of parasitemia were markedly different between NB and adult mice. First, the onset and resolution of parasitemia were significantly delayed. Next, we observed that the percentage of parasitemia was much higher in NB mice than in adult mice. The magnitude and the kinetics of parasitemia observed in the NB mouse model parallel what occurs in infants despite the fact that infants in endemic areas likely have circulating maternal antibodies against *Plasmodium* while *P. yoelii* infected NB mice do not. As such, this model could be used to study the mechanisms and outcomes of malaria latency in infants.

### Protective effect of a milk-based diet on parasitemia

**Figure 2:**
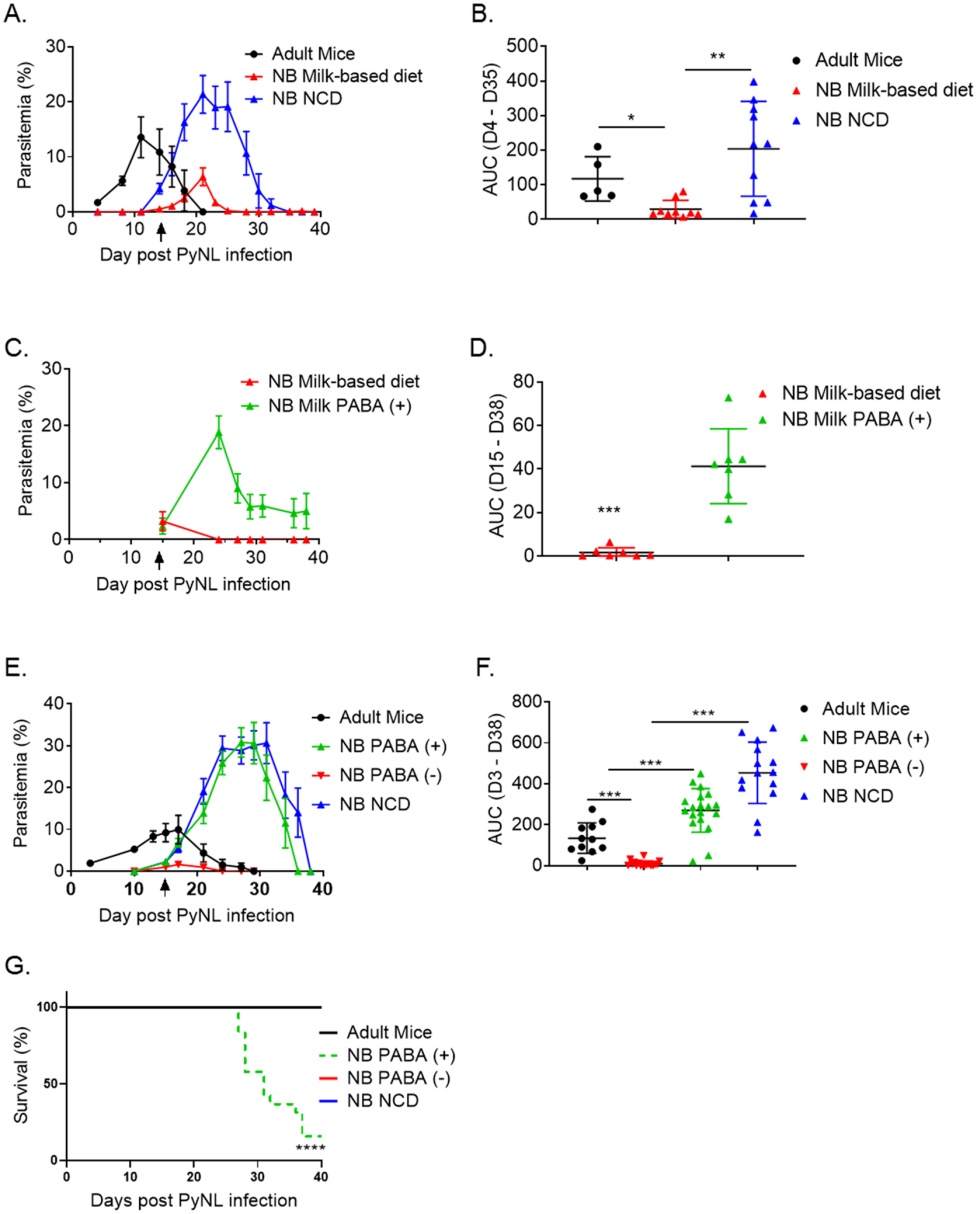
Parasitemia levels decrease when weanlings are fed with a milk-based diet or PABA-deficient diet. (**A**) Two groups of 6- to 7-days old NB and 7- to 8-weeks old adult C57BL/6 mice were infected with 1 × 10^6^ PyNL parasites. Five adult mice were fed with NCD throughout the experiment. At the weaning time point (day 21 post birth, black arrow), one group of weanlings were fed with milk-based diet for 2 weeks, and the other group with NCD. The number of mice in the milk-based diet and the NCD groups were 11 and 10, respectively. Parasitemia levels were assessed by blood smears on indicated time points until clearance. (**B**) AUC of parasitemia burden between days 4 to 35 post infection shown in panel A are plotted. (**A**, **B**) Cumulative data (mean ± SEM) from two experiments are shown. (**C**) Two groups of 6- to 7-days old NB mice, each group containing 7 mice, were infected with 1 × 10^6^ PyNL parasites. NB mice were weaned at day 21 post birth (black arrow) and were fed with milk-based diet or milk-based diet containing PABA (PABA (+)) for 23 days. Parasitemia levels were evaluated by blood smears on indicated time points. (**D**) AUC of parasitemia burden between days 15 to 38 post infection shown in panel C are plotted. (**C**, **D**) Data (mean ± SEM) from one experiment is shown. (**E**) Three groups of 6- to 7-days old NB mice were infected with 1 × 10^6^ PyNL parasites. NB mice were weaned at day 21 post birth (black arrow) and were fed with PABA-containing (PABA (+)), PABA-deficient (PABA (-)) or NCD. Parasitemia levels were evaluated by blood smears on indicated time points until clearance. (**F**) AUC of parasitemia burden between days 3 to 38 post infection shown in panel E are plotted. (**E**, **F**) Cumulative data (mean ± SEM) from two experiments containing 19, 17, 14 and 12 mice in PABA (+), PABA (−), NCD, and adult mice groups, respectively, are shown. Student’s t-test was used to compare the AUC between groups. *p < 0.05, **p < 0.01, and *** p < 0.001. (**G**) The survival of mice in panel E was assessed until 40 days after infection. Results are expressed as survival curves analyzed using the Log-rank (Mantel Cox) test for 12 PABA (+), for 10 PABA (−) and for 10 NCD mice. ****p < 0.0001 indicates comparison of survival between PABA ( +) group and the mice in the other groups (PABA (-), NB NCD and Adult mice).

The long sub-latent period of undetectable parasitemia in the NB mice is similar to what occurs in malaria infections in infants. In humans, it was previously thought that infants were rarely infected with *Plasmodium* parasites, until PCR-based diagnostics revealed a large population of asymptomatic but sub-clinically infected infants under 6 months of age^28^. The emergence of *P. yoelii* 2 weeks after the challenge suggests that NB mice also harbor low levels of parasitemia that is not detectable by Giemsa staining of blood smear. An important characteristic of the NB mouse parasitemia kinetics was that weanlings became smear positive right after they were switched from maternal milk to NCD after weaning at 21 days of age (Fig. 1A,C). To test whether maternal milk prevented the establishment of infection during the pre-weaning period, we restricted weanlings to milk-based diet after weaning. Two groups of 6- to 7-days old mice and adult mice were infected with *P. yoelii* parasite as in Fig. 1. After weaning the mice at day 21 post birth, one group was kept on a milk-based diet, and the other group (the control group) received NCD. Parasitemia were monitored until the mice cleared infection. Adult mice and the NB control group parasitemia kinetics and magnitude were the same as those observed in previous challenge experiments (Fig. 2A). Parasitemia was detected in NB control group after the weanlings were switched to NCD and the peak parasitemia percentage and AUC (Fig. 2A,B) were higher than the adult mice levels. When the infection in the two weanling groups were compared, the kinetics of parasitemia was similar between the groups (Fig. 2A). Mice in both the groups were blood smear positive after they were weaned and their peak parasitemia was at day 21 post infection. However, the peak parasitemia in the group fed with milk-based diet was nearly 4 times less than in the group fed with NCD (6.3% vs 21.36%) (Fig. 2A). Parasitemia burdens quantified as AUC until day 35 showed a significant difference between the NCD and milk-based diet fed weanlings (Fig. 2B). Moreover, the group fed with milk-based diet cleared parasitemia noticeably earlier (by day 25 post infection) than the NCD group (by day 35 post infection). Thus, replacement of NCD with a milk-based diet was sufficient to significantly diminish *P. yoelii* infection in weanlings.

### Protective effect of a PABA-deficient diet on parasitemia

As shown previously with adult rodent and monkey studies^23,24,29^, our NB mouse study also demonstrated that a milk-based diet severely reduces malaria parasitemia. Milk is deficient in PABA^22^, a dietary molecule required for folate biogenesis by *Plasmodium*^25^. In adult mice, removal of PABA from diet protects mice from the establishment of malaria infection while supplementation of PABA-deficient diet with PABA restores the infection^24,30^. To test whether the absence of PABA in milk prevented *P. yoelii* to establish parasitemia in weanlings, we challenged NB mice at 6 to 7 days of age and fed weanlings with a milk-based diet supplemented with PABA after weaning (at 21 days of age) and compared parasitemia to those fed with milk-based diet. Confirming the role for PABA in malaria propagation, peak parasitemia levels (18.87%) in mice fed with PABA containing milk-based diet were 5.6 times more than those fed with milk-based diet without PABA (Fig. 2C). Parasitemia burdens quantified as AUC until day 38 showed a significant difference between the milk-based diet fed weanlings and those fed with milk-based diet supplemented with PABA (Fig. 2D). Interestingly, in addition to manifesting high parasitemia, 3 out of 7 mice in the group fed with PABA containing milk-based diet died on days 29, 36, and 38 after parasite challenge (Supplemental Fig. 1A). Mice fed with PABA containing milk-based diet also tended to weigh less although the weight difference between the two groups did not reach statistical significance (Supplemental Fig. 1B).

Next, we investigated the significance of PABA using diets other than milk. For this purpose, we challenged three groups of NB mice with *P. yoelii* and fed them after weaning (at 21 day of age) with a diet containing PABA (NB PABA (+) group), the same diet deficient in PABA (NB PABA (−) group) or NCD (NB NCD group). Adult mice on NCD were also challenged with *P. yoelii*. Malaria infection was monitored until parasitemia clearance. We found that, as with milk-based diet group (Fig. 2C), weanlings fed a PABA-deficient diet controlled parasitemia (Fig. 2E,F). Peak parasitemia for this group was 1.6% on day 17 (3 days after weaning) post challenge, which was resolved by day 24 post challenge. In comparison, peak parasitemia (day 27 post challenge) level for the weanlings fed with the same food but containing PABA was nearly 20 times more (30.77%) than the weanlings fed with PABA-deficient food (Fig. 2E,F). This group remained parasitemia positive until day 36 post challenge. The magnitude and the kinetics of parasitemia in weanlings fed with NCD were almost identical to the group fed with PABA-supplemented diet (Fig. 2E,F). As with mice fed with PABA containing milk diet (Fig. 2C), some of the mice fed with PABA-supplemented regular diet died after *P. yoelii* challenge. Despite manifesting comparable levels of parasitemia, 16 out of 19 (84%) mice in PABA containing diet fed group died by day 37 post-challenge while all the weanlings fed with NCD survived (Fig. 2G). Also resembling the outcome in mice fed with PABA containing milk diet, mice fed with PABA-supplemented regular diet weighed less than the mice fed with PABA-deficient diet as well as the mice fed with NCD (Supplemental Fig. 2A). The death of mice fed with PABA containing diet was not related to anemia because hemoglobulin, red blood cells (RBC) and hematocrit values for mice fed with NCD and PABA-supplemented diet were comparable (Supplemental Fig. 2B–D). Although inability to access food and water may have contributed to weight the loss and the death toll in PABA-deficient diet fed mice, the exact cause of mortality in mice fed with PABA containing food remains unknown and needs to be explored further. Nevertheless, diet experiments indicated that the absence of PABA in maternal milk may be preventing the establishment of *P. yoelii* infection until the weaning of mice. Furthermore, continued PABA deprivation of parasite with a diet deficient in PABA after the weaning prevents the rapid increase in parasitemia observed in mice fed with NCD after weaning. Conversely, supplementation of milk with PABA allows the rapid propagation of *P. yoelii* in weanlings.

### Kinetics of Tfh cells, germinal center (GC) B cells, and plasma cells in PyNL infected NB and adult mice

**Figure 3:**
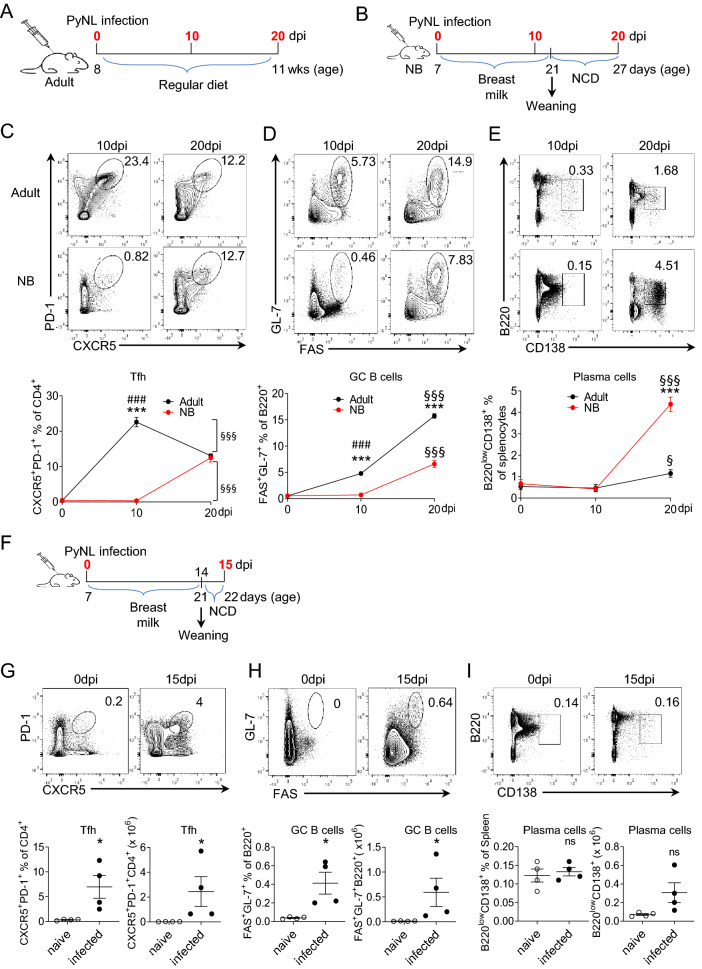
NB mice Tfh, GC B cell and plasma cell development coincides with the switch to NCD after weaning. Six to 7-days old NB and 8 to 12-weeks old adult C57BL/6 mice were infected (i.p.) with 1 × 10^6^ PyNL parasites. (**A**) Splenocytes of adult mice were analyzed in flow cytometry on days 0, 10 and 20 after parasite challenge (days post infection, dpi). (**B**) Splenocytes of NB mice were analyzed 0, 10 and 20 days after parasite challenge at 7 days after birth. NB mice remained on breast feeding until 21 days of age when they were switched to NCD. (**C**–**E**) Representative dot plots depict the percentage of splenic PD-1^+^CXCR5^+^ (Tfh) cells on CD4^+^ pre-gated T cells (**C**), GL-7^+^FAS^+^ (GC B cells) on B220^+^ pre-gated B cells (**D**) and B220^low^CD138^+^ (plasma cells) on pre-gated live cells (**E**) at indicated days post infection. Formation and resolution kinetics of Tfh cells, GC B Cells, and plasma cells were plotted as mean percentage of cells. Results are expressed as mean ± SEM (n = 4); *p < 0.05, and ***p < 0.001 indicate adult vs NB mice; ^###^p < 0.001 indicates 0 dpi vs 10 dpi in both adult and NB mice, and ^§§§^p < 0.001 indicates 10 dpi vs 20 dpi in both adult and NB mice. (**F**) Splenocytes of NB mice were analyzed 0 and 15 days after PyNL challenge at 7 days after birth. NB mice remained on breast feeding until 21 days of age when they were switched to NCD. (**G**–**I**) Representative dot plots depict the percentage and cell number of splenic PD-1^+^CXCR5^+^ (Tfh) cells on CD4^+^ pre-gated T cells (**G**), GL-7^+^FAS^+^ (GC B cells) on B220^+^ pre-gated B cells (**H**) and B220^low^CD138^+^ (plasma cells) on pre-gated live cells **(I)** at indicated days post infection. Formation and resolution kinetics of Tfh cells, GC B Cells, and plasma cells were plotted as mean percentage and cell numbers of cells. Results are expressed as mean ± SEM (n = 4); *p < 0.05 indicate naive vs infected. One out of two experiments with similar results is shown.

In *P. yoelii* infection, the resolution of infection is associated with the production of anti-malarial-antibodies which is dependent on optimum Tfh and GC B cell responses^27^. We therefore investigated the splenic Tfh, GC B cell and plasma cell development in *P. yoelii* infected adult and NB mice on days 10 and 20 after parasite challenge (Fig. 3A,B; Supplemental Fig. 3). Consistent with previous observation^27^, in adult mice the percent of CXCR5^+^PD-1^+^ Tfh cells among CD4 T cells peaked earlier than B220 + GL-7 + FAS + GC B cells did (Fig. 3C,D). In adult mice, Tfh cells were measured highest at day 10 and GC B cells were measured highest at day 20 post infection. Adult mice plasma cells were first detected at day 20 (Fig. 3E). In NB mice, Tfh, GC B cells and plasma cells did not develop at day 10 after challenge when parasitemia levels were very low (Fig. 3C–E). By day 20, coinciding with the increase in parasitemia, weanling Tfh, GC B cells and plasma cells were significantly elevated compared to day 10 (p < 0.001). Interestingly, the percentage of splenic plasma cells were significantly higher (p < 0.001) in weanlings than in adult mice (Fig. 3E). As reported previously^31^, the numbers of Tfh and GC B cells in NB and weanlings were significantly lower than the adult cells, proportional to the differences in total splenocytes, CD4 + T cells and B cells between the two age groups (Supplemental Fig. 4). It is important to note that the frequencies of all three cell subsets increased only after the mice were weaned 21 days after birth (14 days after challenge) and switched to NCD (Fig. 3C–E).

To further investigate the relationship between the onset of GC reaction, the switch to NCD and the increase in parasitemia, we repeated the NB *P. yoelii* challenge experiment and measured the frequencies of Tfh, GC B cells and plasma cells one day after (15 days after malaria challenge) the weaning of mice (Fig. 3F). Although not as high as the levels at day 20, both Tfh and GC B cell frequencies and the numbers were significantly elevated compared to naïve mice levels (Fig. 3G,H). The frequency of plasma cells was not different than those of naïve mice (Fig. 3I). Thus, the development of GC reaction and the emergence of plasma cells closely paralleled the parasitemia kinetics (Fig. 1A,C) in both adult and NB mice challenged with *P. yoelii*.

### Robust activation of the immune system in weanlings fed with PABA-deficient diet

**Figure 4:**
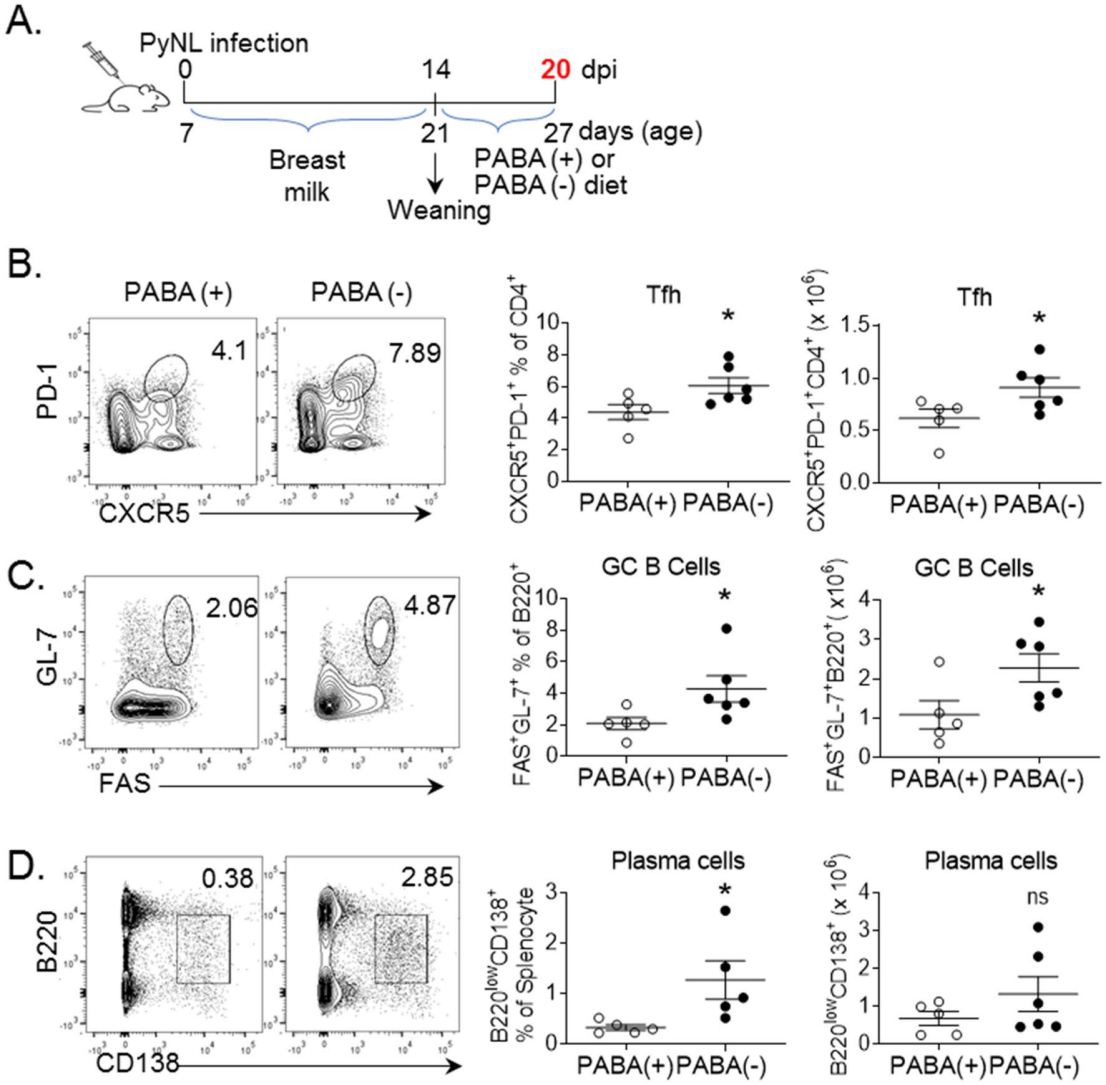
Mice fed with PABA negative diet develop Tfh cells, GC B cells and plasma cells despite experiencing low parasitemia. (**A**) Groups of 6- to 7-days old NB mice were infected (i.p.) with 1 × 10^6^ PyNL parasites and splenic Tfh cells, GC B cells and plasma cells were analyzed at 20 dpi. NB mice remained on breast feeding until 21 days of age when they were switched to PABA-containing or PABA-deficient diet. Mice in both groups were sacrificed at 20 dpi and splenocytes were analyzed in flow cytometry. Representative dot plots depicting the percentage and cell numbers of splenic PD-1^+^CXCR5^+^ (Tfh) cell on CD4^+^ pre-gated T cells (**B**), GL-7^+^FAS^+^ (GC B cells) on B220 pre-gated B cells (**C**) and B220^low^CD138^+^on pre-gated live cells (**D**) and the accompanying mean percentages and cell numbers were plotted. One out of two experiments with similar results is shown. Results are expressed as mean ± SEM (n = 5); *p < 0.05 indicates PABA + vs PABA- diet fed mice.

Next, we investigated the host response in *P. yoelii* infected weanlings fed with PABA-containing or PABA-deficient diet to assess whether dramatically different levels of parasitemia in weanlings fed with PABA-deficient and PABA-containing diet (Fig. 2C,E) alters the GC reaction. Groups of 6- to 7-days old mice were infected with *P. yoelii* and weanlings were given PABA-containing and PABA-deficient diet when mice were weaned at 21 days after birth (Fig. 4A). The development of Tfh cells, GC B cells and plasma cells were analyzed at 27 days after birth (20 dpi). Interestingly, despite manifesting low parasitemia (Fig. 2E), mice fed with PABA-deficient food had significantly higher frequencies of Tfh cells, GC B cells and plasma cells as compared to the mice fed with PABA-containing diet (Fig. 4B–D). The numbers of Tfh cells, and GC B cells were also higher in PABA-deficient diet fed weanlings than those fed with PABA-containing (Fig. 4B,C). At the same time, although weanlings fed with NCD had comparable levels of parasitemia with mice fed with PABA-containing diet (Fig. 2E), the frequencies of Tfh cells, GC B cells and plasma cells were much lower in PABA-containing diet fed mice (Fig. 4B–D) than NCD fed mice (Fig. 3C–E). These results indicated that despite very low parasitemia, weanlings fed with PABA-deficient diet were able to mount a robust GC reaction.

### Weanlings fed with a PABA-deficient diet are protected against a second PyNL challenge

**Figure 5:**
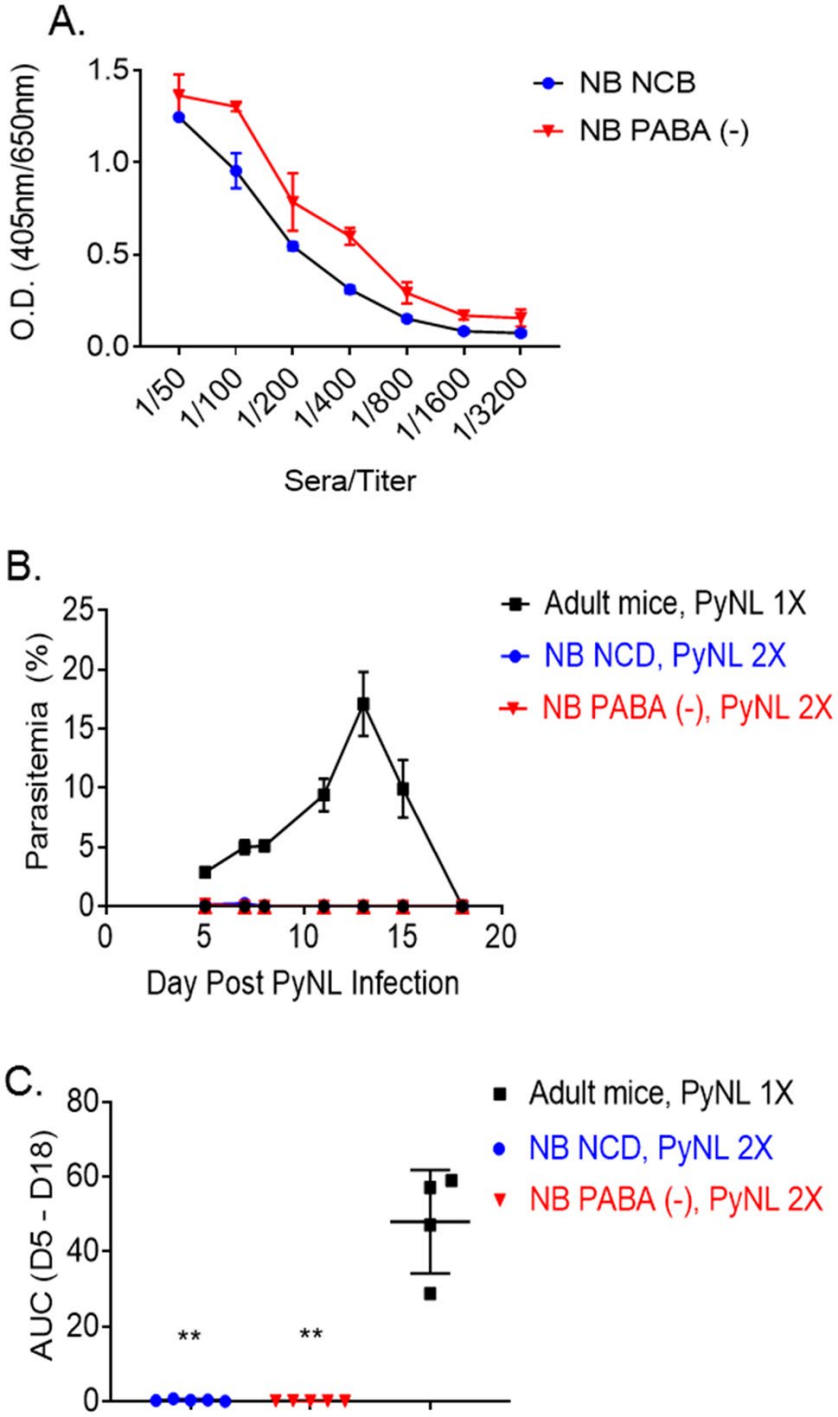
*P. yoelii* infected weanlings fed with PABA-deficient diet mount anti-*Plasmodium* antibodies and are protected from a second PyNL-challenge. (**A**) Three *P. yoelii* infected NB mice from each of the groups that were fed with NCD and PABA (-) diet after weaning in Fig. 2E were euthanized two months after the clearance of parasitemia and serum anti-rMSP-1 antibody levels were measured in ELISA. (**B**) The remaining 5 NB mice from each of the groups that were fed with NCD and PABA (-) diet after weaning in Fig. 2E were challenged 2 months after the clearance of parasitemia with 1 × 10^6^ PyNL parasites a second time and parasitemia levels were assessed by blood smear on indicated time points. As a control, 5 malaria naïve adult mice were also challenge with 1 × 10^6^ PyNL parasites. Results are expressed as mean % parasitemia ± SEM. **C**) AUC of parasitemia burden between days 5 to 18 post infection shown in panel B are plotted as mean AUC ± SEM. Student’s t-test was used to compare the AUC between groups. **p < 0.01. Experiment was performed once.

Feeding weanlings with PABA-deficient diet indicated that these mice mount a robust Tfh cell, GC B cell and plasma cell responses despite experiencing very low parasitemia. We next assessed whether the robust Tfh cell and GC B cell responses render the PABA-deficient diet fed weanlings resistant to a second PyNL challenge. To address this question, we rechallenged the weanlings fed with NCD (NB NCD) and PABA-deficient (NB PABA (-)) diet in Fig. 2E with *P. yoelii* and assessed the infection outcome. Prior to re-infection of mice two months after the clearance of parasitemia, we measured serum anti-rMSP-1 antibody responses to evaluate whether the development of Tfh cell, GC B cell and plasma cell responses resulted in the production of anti-*Plasmodium* antibodies. We found comparable levels of antibody responses between the two groups despite limited parasitemia in PABA-deficient diet fed mice (Fig. 5A). Next, both groups of mice were rechallenged with a second dose of 1 × 10^6^ PyNL parasites. Age matched naïve mice were also challenged with PyNL as control. Regardless of the type of diet mice were fed post weaning period, they were fully protected from a second PyNL challenge, while naïve mice had typical magnitude and kinetics of parasitemia (Fig. 5B,C). These results indicated that severely reduced parasitemia mediated by PABA-deficient diet is sufficient to induce protective immune response against a second PyNL exposure.

### Removal of PABA from diet rapidly decreases parasitemia in *P. yoelii* infected weanlings

**Figure 6:**
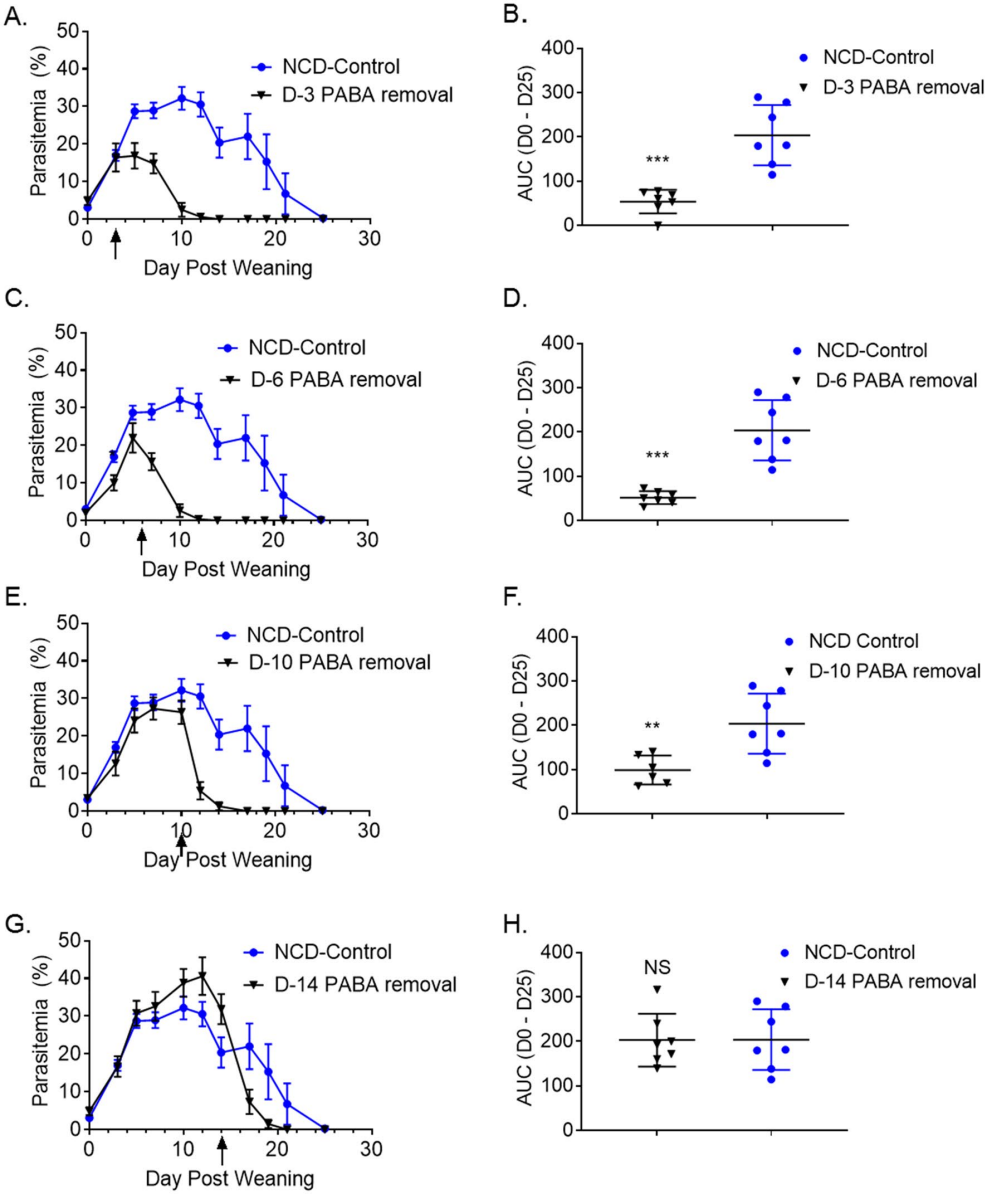
Removal of PABA from the diet of *P. yoelii* infected weanlings sharply reduces parasitemia. Five groups of 6- to 7-days old NB mice, each containing 7 mice, were infected with 1 × 10^6^ PyNL parasites. All mice were transferred to NCD after weaning at day 21 post birth. Subsequently, groups of mice were switched to PABA-deficient diet at 3 (**A**), 6 (**C**), 10 (**E**) and 14 (**G**) days post weaning. One group stayed on NCD. Parasitemia levels were assessed by blood smears on indicated time points until clearance. Results are expressed as mean % parasitemia ± SEM for 7 mice per group. (**B**, **D**, **F**, and **H**) AUC of parasitemia burden between days 0 to 25 post infection shown in panels A, C, E and G are plotted as mean AUC ± SEM in panels B, D, F and H, respectively. Student’s t-test was used to compare the AUC between groups. **p < 0.01, ***p < 0.001. Experiment was performed once.

The requirement for PABA in *Plasmodium* growth is well documented^32^. Our studies with milk-based diet and PABA-deficient diet clearly demonstrated that challenging of weanlings that are already on PABA-deficient diet prevents the establishment of infection to a high degree. We next sought to understand whether removal of PABA in weanlings after the establishment of infection alters the course of parasitemia. For this purpose, groups of NB mice were infected at 6 to 7 days of age. All mice were put on NCD after weaning. To test the effect of PABA-deficient diet on established infection, groups of mice were switched from NCD to PABA-deficient diet on days 3-, 6-, 10- and 14-days post weaning. Parasitemia percentages and AUC quantifications in each group were compared to control group of weanlings that were kept on NCD after weaning. Other than the group that received PABA-deficient diet 14 days after weaning (Fig. 6G,H), all groups benefitted from switch to PABA-deficient diet. Removal of PABA from diet 3 days after weaning led to lower percentage of parasitemia (16.91%) compared to mice kept on NCD (28.7%) 2 days after (5 days after weaning) the switch in diet (Fig. 6A). Also, mice in this group cleared parasitemia markedly earlier than those kept on NCD (Days 14 vs 25 after weaning). Mice that began receiving PABA-deficient diet 6 days after weaning also manifested a rapid decrease in parasitemia. One day after the diet change, their parasitemia levels (15.7%) were significantly lower than those kept on NCD (28.9%) (Fig. 6C). These mice were also parasitemia-free 14 days after weaning. Introduction of PABA-deficient diet 10 days after weaning resulted in a significant decrease in parasitemia 2 days later compared to NCD-fed mice (Fig. 6E). These mice cleared parasitemia 7 days before the NCD-fed mice. Parasitemia burdens quantified as AUC until day 25 showed a significant difference between the NCD-fed mice and those that received PABA-deficient diet 3 (Fig. 6B), 6 (Fig. 6D), and 10 (Fig. 6F) days after weaning. The beneficial effect of removing PABA from diet against *P. yoelii* infection has been shown by Kicska and colleagues in adult mice also^24^. They reported that parasitemia levels dropped and mice survived after the introduction of PABA-deficient diet 3 days post challenge with the lethal *P. yoelii* strain, YM. Collectively, our results, together with the study by Kicska and colleagues indicate that switch to PABA-deficient diet is beneficial against malaria even after the infection is established.

## Discussion

Although the incidence of clinical malaria has been considered as rare during the first year of life^3,4,33,34^, improvements in molecular diagnostic methods indicate that infants are frequently infected without symptoms or present with atypical symptoms^3,7,35^. Here, we established a NB mouse malaria model, the results of which suggest that the subclinical manifestation of malaria during infancy may be due to protection afforded by the PABA-deficient maternal milk.

The exact reasons for the relative protection of infants from severe malaria are not known. The transfer of anti-malarial antibodies through the placenta and milk have been proposed as a possible factor in preventing the establishment of infection during infancy^20,21,36,37^. The delay in the onset of parasitemia in our experiments cannot be attributed to maternal antibodies because the dams were naïve to *P. yoelii*. Although early studies suggested the prevalence of HbF during neonatal period as another possible reason in the protection of infants from *P. falciparum* infection^16^, subsequent studies demonstrated that cord RBCs can be infected with *P. falciparum*^18,19^. Moreover, HbF cannot account for the suppressed parasitemia in NB mice because, unlike humans, mice RBCs do not have HbF^38^.

The NB mouse *P. yoelii* challenge model presented in this study recapitulates the human infant malaria as the *P. yoelii* challenged 6- to 7-days old mice were blood smear negative until weaning while the adult mice were parasitemia positive as early as 2 days post challenge. The delay in the establishment of parasitemia in NB mice appears paradoxical because in general, early age is associated with increased susceptibility to infectious diseases due to the inability of neonates to mount efficient immune response against microbial antigens^39–41^. Using a range of diets, we have observed that the delay in the establishment of parasitemia until the mice are weaned can be a direct result of the PABA-deficient milk-based diet in suckling newborns.

The requirement for exogenous PABA in *Plasmodium* propagation has been recognized since 1930s^42^. PABA is involved in the synthesis of folate, a member of the vitamin B family with essential functions ranging from purine biosynthesis to protein and DNA methylation^43^. Although *Plasmodium* has the machinery for de novo PABA synthesis, this pathway is only active to maintain parasite survival when dietary PABA is scarce, such as in milk^29,32^. The protection afforded by a milk-based diet against malaria has been first shown in *P. berghei* infected rats and mice^29^. A similar limitation of *P. cynomolgi* infection has also been shown in monkeys kept on a milk-based diet^44^. Subsequently, Hawking DM demonstrated that mice can be infected with *P. berghei* if the milk-based diet is supplemented with PABA^22^. In an attempt to demonstrate the effect of milk on NB rats’ and young monkeys’ susceptibility to malaria, Hawking infected suckling NB rats and baby monkeys with *P. berghei* and *P. cynomolgi*, respectively^23^. However, in these experiments, the suckling rats and monkeys were protected from *Plasmodium* challenge when dams were fed with a diet deficient in PABA and neonates born to dams fed with NCD were not challenged.

A more comprehensive study conducted in adult mice by Kicska and colleagues, established the role of PABA in lethal (YM) and non-lethal (17X) *P. yoelii* infection^24^. Similar to what we have observed in NB mice fed with milk-based diet or PABA-deficient diet before *P. yoelii* challenge or after the establishment of infection, they also found that *Plasmodium* cannot establish infection when PABA is deficient in host diet. Another similarity between our study in NB mice and their study in adult mice is that both age groups were able to resist a second challenge after the clearance of parasitemia in PABA-deficient diet fed mice. We extended these observations by investigating the GC reaction in neonates born to mothers fed with NCD. Paralleling the kinetics of parasitemia, the development of Tfh cell and GC B cells started right after the weaning of mice when parasitemia started to increase. We also found that weanlings given PABA-deficient diet mounted robust Tfh cell, GC B cell and plasma cell responses despite experiencing very low parasitemia. More importantly, these mice were fully protected from a second PyNL challenge 2 months after the recovery from the first infection, as did the NCD-fed weanlings. The protection was likely due to anti-*Plasmodium* antibody responses because they had comparable serum anti-rMSP-1 antibody levels with the NCD-fed mice after the clearance of parasitemia.

Collectively, the NB *P. yoelii* infection model study underscores the importance of maternal milk in preventing the establishment of *Plasmodium* infection in early age. Although we were not able to devise a method to feed the NB mice with PABA-supplemented milk prior to weaning, experiments with PABA-supplemented milk and PABA-deficient diet after weaning of mice point to the deficiency of PABA in milk as the likely reason for the protection of suckling mice from malaria infection. These experimental data provide a plausible explanation for the clinical observations where well-fed children are shown to experience more severe malaria^45^ and exclusive breastfeeding helps reduce the risk of clinical malaria^20^. Importantly, the suppression of malaria by depriving *Plasmodium* from dietary PABA does not prevent host from mounting a protective immune response. Our data also suggest that limiting PABA intake may even have a therapeutic effect in neonates with established malaria infection. Thus, further studies are warranted to investigate the value of dietary PABA restriction in malaria prevention as well as therapeutics.

## Materials and methods

### Mice and *P. yoelii* challenge

Seven to 8-weeks old C57BL/6 female and male mice (Jackson Laboratory, Bar Harbor, ME) were paired to obtain NB mice. Seven-day old (both sexes) or 7 to 8-weeks old adult female C57BL/6 mice were used for *P. yoelii* NL (PyNL) challenge. NB mice were weaned at 21 days post birth from their mothers. Studies were conducted under the guidance for the care and use of laboratory animals specified by the National Institute of Health (Bethesda, MD). Experimental procedures were approved by the Institutional Animal Care and Use Committee of the Center for Biologics and Research (FDA) under Animal Study Protocol 2008–08.

### PyNL infection

PyNL infected RBCs were stored as frozen stocks and used to infect C57BL/6 mice by the intraperitoneal (i.p.) route. When approximately 10% parasitemia levels were observed by examining Giemsa blood smears, blood was collected and diluted in PBS to achieve a concentration of 1 × 10^6^ parasites/200 μl to infect adult mice as per previous studies^27^, or 1 × 10^6^ parasites/30 μl to infect 7-days old NB mice. Percent parasitemia (parasitized RBCs/total RBCs × 100) was measured starting at day 3–5 post-infection and until parasitemia cleared.

### Diets

After mice were weaned at day 21 post birth, they were fed with ProLab Isopro RMH 3000 normal chow diet (NCD) (LabDiet, St. Louis, MO), milk-based diet, milk-based diet supplemented with PABA, PABA-deficient diet (Envigo Teklad Diets, Madison, WI), or PABA-supplemented diet (Envigo). The milk-based diet consists of milk pellets and a potable milk solution. Milk pellets were prepared by adding 0.5 ml of sterile water to 5 gr of Carnation Instant Non-fat milk powder (ThermoFisher, Waltham, WA). The mixture was stirred until a sticky dough-like substance formed, which was left for drying to obtain the pellets. For the preparation of milk solution, we added 18.0 g of milk to 100 ml of autoclaved water. Pellets were changed every 2–3 days for mice undergoing a milk-based diet, and the potable milk was changed daily. When preparing a milk-based diet containing PABA, 0.0375 g of PABA was diluted into 25 ml sterile water to achieve 0.0015 g/ml PABA solution. Next, 0.5 ml of this PABA solution was added to 4.5 g of milk powder to achieve 150 µg PABA per 1 g of milk. Pellets were prepared as described for milk-based pellets. Some mice were fed with either the NCD (ProLab Isopro), PABA-deficient diet (Envigo), or the PABA-deficient diet supplemented with 150 µg/g of PABA (Envigo) after weaning (21-day-old). The amount of PABA in PABA-supplemented diet was selected based on the report by Kicska et al.^24^ who also used NCD supplemented with 150 µgr/gr of PABA when assessing the effect of PABA on adult mouse response to *P. yoelii* infection.

### Measurement of anti-PyNL antibody responses

Serum samples were collected from C57BL/6 mice at 2-months post clearance of PyNL infection to measure antibody levels using ELISA as described previously^27^. The ELISA plates (ThermoFisher) were coated with 70 ng *P. yoelii* Merozoite Surface Protein 1 (C-terminal 19-kDa fragment [rMSP-1]) in 200 µl of coating buffer (15 mM sodium carbonate and 35 mM sodium bicarbonate, pH 9.6) overnight at 4 °C. After rinsing three times with washing buffer (PBS/0.05% Tween-20), blocking buffer composed of 5% milk (MP Biomedicals, Irvine, CA) in PBS was added to wells for 1 h. Following the blocking step, 100 µl of titrated sera were added to wells at a starting dilution of 1:50. After 2 h of incubation at room temperature, the plates were washed and peroxidase conjugated mouse IgG specific secondary antibodies (Southern Biotechnology Associates, Birmingham, AL) were added for 1 h at room temperature. After washing the plates three more times, 100 µl ABTS substrate (KPL, Gaithersburg, MD) was added for 30 min, and the absorbance was measured at a wavelength 405/650 nm using a VERSA max microplate reader (Molecular Devices, Sunnydale, CA).

### Complete blood count (CBC)

Mouse blood was collected via cardiac puncture using an un-coated needle (Monoject 250 hypodermic 23-gauge needle, Cardinal Health, Laurel, MD) and 1 ml Monoject tuberculin syringe (Cardinal Health, Laurel, MD) into an EDTA-coated tube (Greiner Bio-One, Monroe, NC). Complete blood count was measured using a Heska Element HT5 (Loveland, CO) instrument.

### Flow cytometry

Flow cytometry experiments were performed as described previously^41^. Single cell suspensions were prepared from spleen and dead cells were excluded after staining with fixable efluor 780 (Affymatrix, Santa Clare, CA) 1:1000 diluted in PBS. Cells were washed and stained with fluorophore conjugated antibodies using flow cytometry buffer containing 0.5% FBS, 2.5 mM EDTA in PBS. For T follicular helper cell (Tfh) analysis, cells were stained with antibodies against CD4 (PerCPCy5.5 conjugated clone GK1.55 from Affymatrix), PD-1 (PE conjugated clone 29F.1A12 from BioLegend, San Diego, CA), and CXCR5 (biotin conjugated clone 2G8 from BD Biosciences, San Jose, CA) were used. Biotin was detected with 1:500 streptavidin-BV421 (BioLegend). For GC B cell analysis, antibodies against B220 (BV605 conjugated clone RA3-6B2 from BioLegend), FAS (APC conjugated clone J02 from BioLegend), T/B cell activating antigen (FITC conjugated clone GL-7 from BioLegend) were used. For plasma cell analysis, antibodies against B220 (APC conjugated clone RA3-6B2 from BioLegend) and CD138 (PE conjugated clone 281–2 from BioLegend) were used. Flow cytometry data were acquired on LSRII flow cytometer (BD Biosciences) and analyzed using the FlowJo software v10 (FlowJo, Ashland, OR).

### Statistical analyses

Graph Pad Prism 7 software was used to analyze the data (Graph Pad Software, San Diego, CA). The antibody ELISA data were evaluated using the ordinary one-way ANOVA test. Survival proportions were analyzed using the Log-rank (Mantel Cox) test. All other statistical evaluations were done using Student’s t test.

## Supplementary Information

Supplementary Information.
