# Supplementary material for: Neonatal mice resist Plasmodium yoelii infection until exposed to para-aminobenzoic acid containing diet after weaning
Source: Sci Rep. 2021 Jan 8;11:90. doi: 10.1038/s41598-020-79703-2 (PMC7794322; doi:10.1038/s41598-020-79703-2)
Supplement: Supplementary file 1 — Supplementary Information. [file 41598_2020_79703_MOESM1_ESM.docx]

**Neonatal mice resist *Plasmodium yoelii* infection until exposed to para-aminobenzoic acid containing diet after weaning**

Marcela Parra, Jiyeon Yang^$^, Megan Weitner^#$^, and Mustafa Akkoyunlu^*^.

*US Food and Drug Administration, Center for Biologics Evaluation and Research, Division of Bacterial Allergenic and Parasitic Diseases, Silver Spring, MD.*

**Supplemental Figures**

**
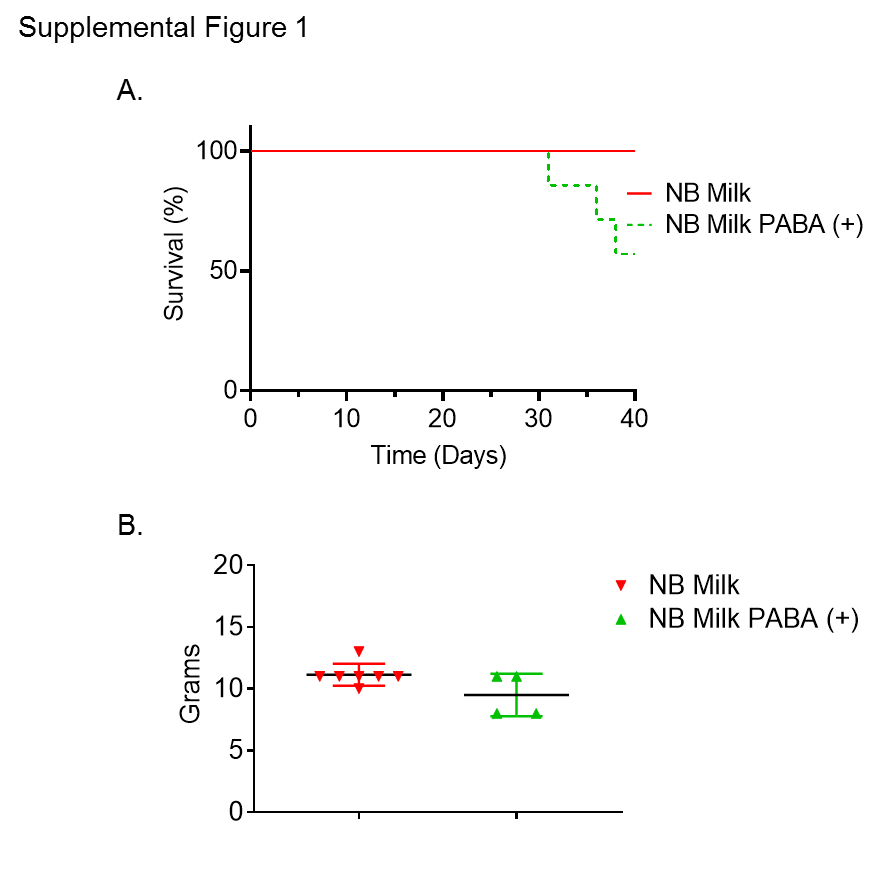
**

**Supplemental Figure 1. The effect of PABA containing milk-based diet on survival and body weight in** ***P. yoelii* infected NB mice. (A and B)** Two groups of 7 6- to 7-days old NB mice were infected with 1 x 10^6^ PyNL parasites. Mice were weaned at day 21 post birth and were fed with a milk-based diet or a milk-based diet containing PABA (PABA (+)) for 24 days. Experiment was performed once. **A)** Survival of mice was assessed until 40 days after infection. Results are expressed as survival curves analyzed using the Log-rank (Mantel Cox) test for 7 weanlings fed with milk-based diet and for 7 weanlings fed with PABA supplemented milk-based diet. **B)** Mice were weighed at day 38 post infection. Results are expressed as mean grams ± SEM for 7 weanlings fed with milk-based diet and for 4 weanlings fed with PABA supplemented milk-based diet. Student’s t test was used for statistical evaluation. Experiment was performed once.

**
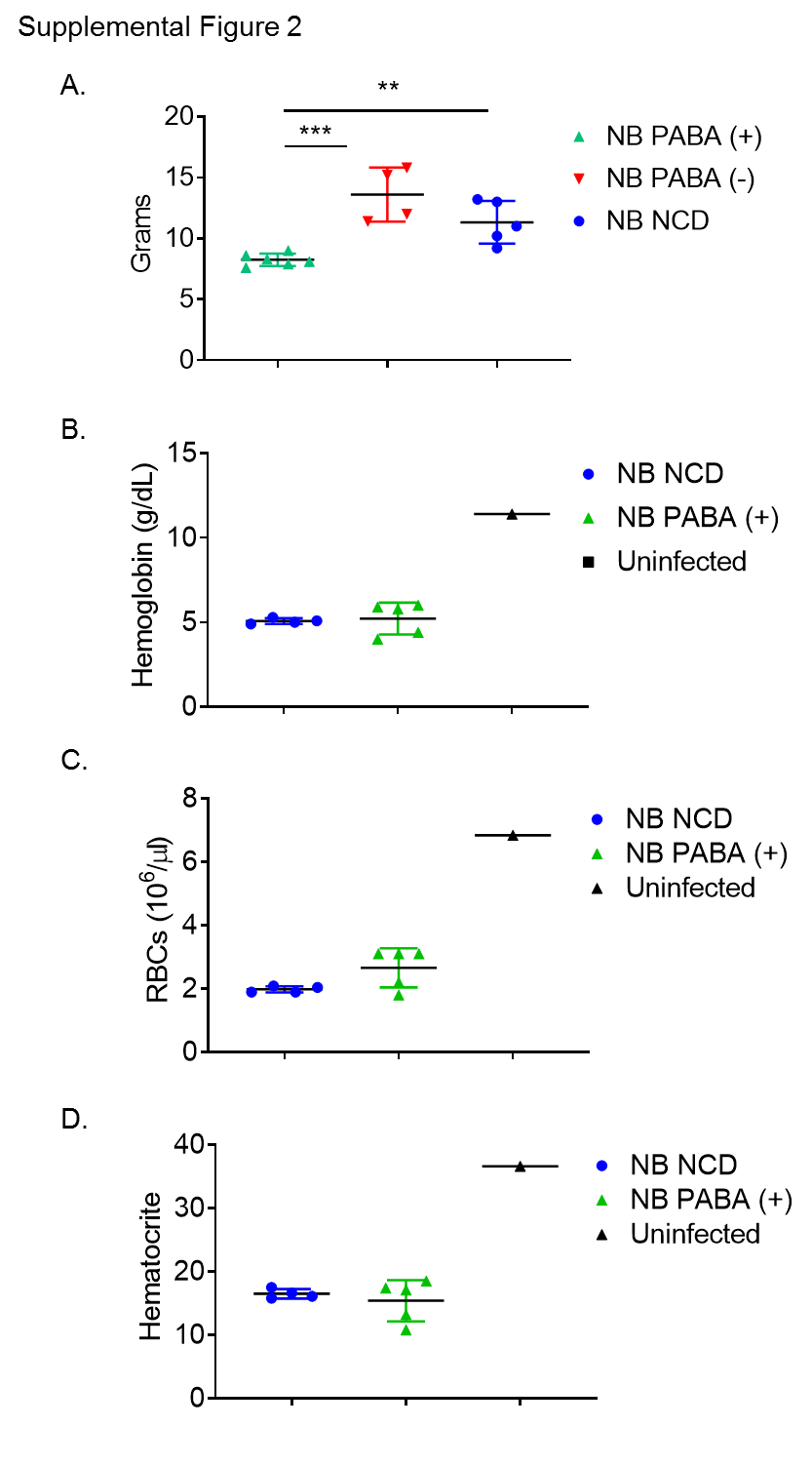
**

**Supplemental Figure 2. Weight and CBC values of *P. yoelii* infected NB mice fed with PABA-deficient diet.** **A)** Three groups of 6- to 7-days old NB mice were infected with 1 x 10^6^ PyNL parasites. At weaning time (day 21 post birth) 6 mice were fed with PABA-containing diet (PABA (+)), 4 mice were fed with PABA-deficient diet (PABA (-)) and 5 mice were fed with NCD. Mice were weighed at day 23 post infection. Results are expressed as mean grams ± SEM. Student’s t test was used for statistical evaluation. **p<0.01 and ***p<0.0001. Two groups of 6- to 7-days old NB mice were infected with 1 x 10^6^ PyNL parasites. Mice were weaned at day 21 post birth and were fed with either PABA-containing (PABA (+)) or NCD. One 28-day old uninfected mouse served as control. Hemoglobin **(B)**, RBC **(C)** and hematocrit **(D)** values were determined at 21 days post infection. Results are expressed as mean ± SEM for 4 to 5 mice per group. Experiment was performed once.

**
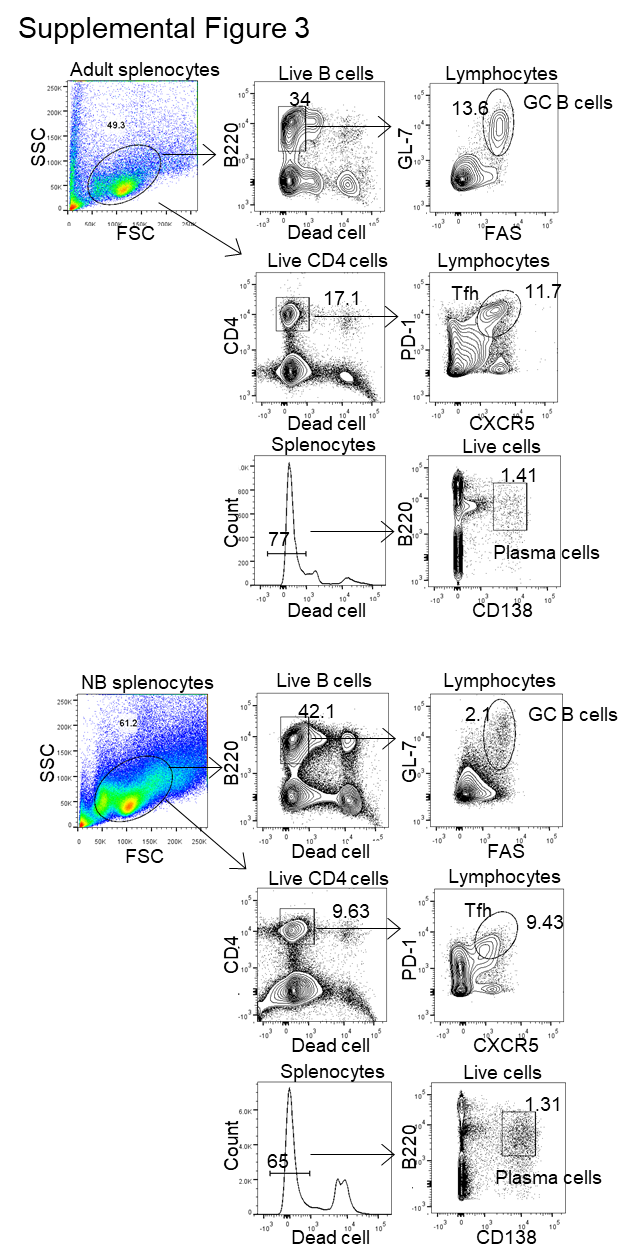
**

**Supplemental Figure 3. Gating strategy for Tfh, GC B cells and Plasma cells.** Adult (7- to 8-week-old) and neonatal (6- to 7-day-old) mice were infected i.p. with 1 x 10^6^ PyNL parasites. Representative gating images of splenic Tfh cells, GC B cells and plasma cells are from mice at 20 days post parasite challenge.

**
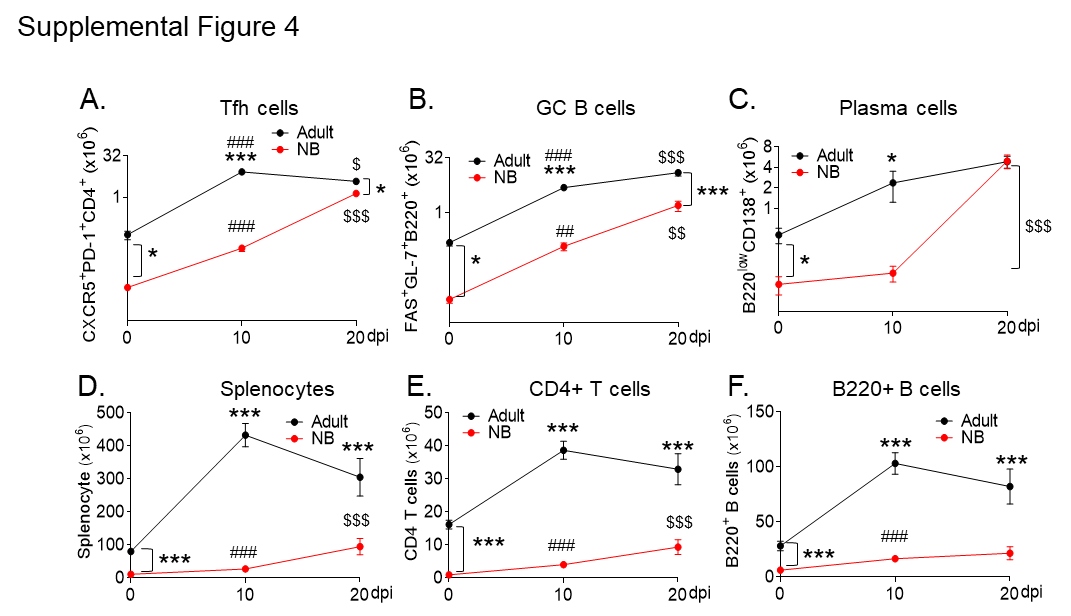
**

**Supplemental Figure 4. Splenic Tfh, GC B cell and plasma cell, CD4 cell, B cell, splenocyte numbers in *P. yoelii* infected NB mice.**

Six to 7-day-old NB and 8- to12-week-old adult mice were infected i.p. with 1 x 10^6^ PyNL parasites. Splenocytes were analyzed in flow cytometry on days 0, 10 and 20 after parasite challenge. Formation and resolution kinetics of CD4^+^PD-1^+^CXCR5^+^ (Tfh cells), B220^+^GL-7^+^FAS^+^ (GC B cells) and B220^low^CD138^+^ (plasma cells) are plotted as cell number per spleen. Results are expressed as mean ± SEM (n=4); *p<0.05, and ***p<0.001 indicate adult vs NB mice; ^##^p<0.01, and ^###^p<0.001 indicate 0 dpi vs 10 dpi in both adult and NB mice, and ^§§§^p<0.001 indicates 10 dpi vs 20 dpi in both adult and NB mice.
